# Supplementary material for: A Scalable Algorithm to Explore the Gibbs Energy Landscape of Genome-Scale Metabolic Networks
Source: PLoS Comput Biol. 2012 Jun 21;8(6):e1002562. doi: 10.1371/journal.pcbi.1002562 (PMC3380848; doi:10.1371/journal.pcbi.1002562)
Supplement: Text S1 — A toy example of the computation of chemical potentials by MinOver with loop correction. (PDF) [file pcbi.1002562.s004.pdf]

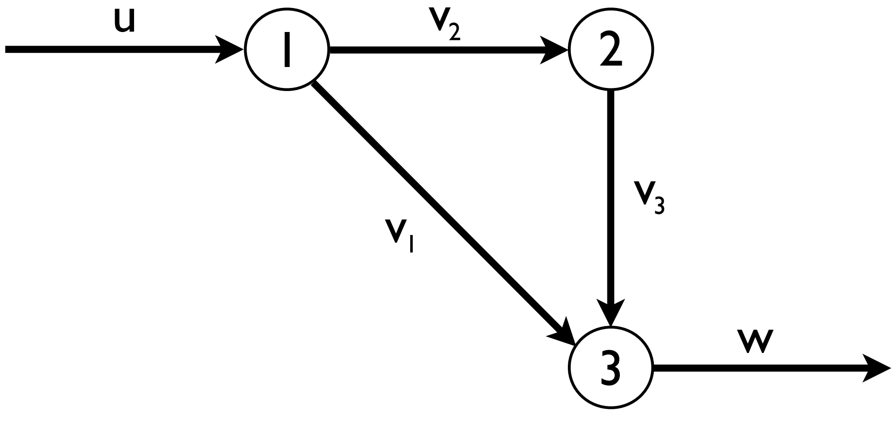

Figure 1. The toy chemical reaction network used in this example: circles denote chemical species and arrows denote reactions.

Supporting Text S1

A toy example of the computation of chemical potentials by MinOver with loop correction

Supporting text for "A Scalable Algorithm to Explore the Gibbs energy Landscape of Genome-scale Metabolic Networks"

Daniele De Martino[1], Matteo Figliuzzi[1], Andrea De Martino[1,2,⋆,†], Enzo Marinari[1,⋆]

1 Dipartimento di Fisica, Sapienza Università di Roma, p.le A. Moro 2, 00185 Roma, Italy
2 CNR-IPCF, Unità di Roma-Sapienza, Roma, Italy
⋆ *These authors contributed equally to this work*
† *E-mail: andrea.demartino@roma1.infn.it*

Note : A computer code implementing the algorithms to compute chemical potentials and identify and remove infeasible loops is downloadable http://chimera.roma1.infn.it/SYSBIO/

We consider the simple reaction network in Fig. 1. By mass-balance, $u = w = v_1 + v_2$ and $v_3 = v_2$. This implies that the following flux configuration satisfies flux balance constraints: $u = w = 1$, $v_1 = -1$ and $v_2 = v_3 = 2$. However v_1 is inverted with respect to the arrow shown in Fig. 1, leading to an infeasible cycle. In specific, if we take $\mu_1 = 10$, $\mu_2 = 3$, $\mu_3 = 5$ (units not specified), then taking unit stoichiometry for simplicity we have $\Delta G_1 = -5$ and $v_1 \Delta G_1 > 0$, violating the second law of thermodynamics. If one applies MinOver to this system with the directions given by the mass-balanced state described above, the algorithm will try to correct chemical potentials in order to reach a solution of the thermodynamic feasibility conditions but will quickly get stuck in the cycle and fail to converge. The output for this case would look as follows (the initial chemical potential vector is $\boldsymbol{\mu} = (10, 3, 5)$):

```
reaction 1 has flux v_1=-1 but the free energy is G_1=-5
from g_1=10 to g_1=9.9
from g_3=5 to g_3=5.1
reaction 1 has flux v_1=-1 but the free energy is G_1=-4.8
from g_1=9.9 to g_1=9.8
from g_3=5.1 to g_3=5.2
[....]
reaction 1 has flux v_1=-1 but the free energy is G_1=-3.2
from g_1=9.1 to g_1=9
from g_3=5.9 to g_3=6
reaction 1 has flux v_1=-1 but the free energy is G_1=-3
from g_1=9 to g_1=8.9
from g_3=6 to g_3=6.1
reaction 3 has flux v_3=2 but the free energy is G_3=1.7
from g_2=4.4 to g_2=4.5
from g_3=6.1 to g_3=6
reaction 1 has flux v_1=-1 but the free energy is G_1=-1.5
from g_1=7.5 to g_1=7.4
from g_3=6 to g_3=6.1
[...]
reaction 2 has flux v_2=2 but the free energy is G_2=0.2
from g_1=5.9 to g_1=6
from g_2=6.1 to g_2=6
reaction 1 has flux v_1=-1 but the free energy is G_1=0
from g_1=6 to g_1=5.9
from g_3=6 to g_3=6.1
reaction 3 has flux v_3=2 but the free energy is G_3=0.1
from g_2=6 to g_2=6.1
from g_3=6.1 to g_3=6
reaction 2 has flux v_2=2 but the free energy is G_2=0.2
from g_1=5.9 to g_1=6
from g_2=6.1 to g_2=6
reaction 1 has flux v_1=-1 but the free energy is G_1=0
from g_1=6 to g_1=5.9
from g_3=6 to g_3=6.1
reaction 3 has flux v_3=2 but the free energy is G_3=0.1
from g_2=6 to g_2=6.1
from g_3=6.1 to g_3=6
reaction 2 has flux v_2=2 but the free energy is G_2=0.2
from g_1=5.9 to g_1=6
from g_2=6.1 to g_2=6
reaction 1 has flux v_1=-1 but the free energy is G_1=0
from g_1=6 to g_1=5.9
from g_3=6 to g_3=6.1
[....]
```

One sees that the time series of the least satisfied constraint ends up cycling from v_1 (initial), to v_3 to v_2 and back to v_1. While running, the algorithm will record this time series. After failing to converge for a large number of time steps, it will finally stop and start searching for loops among the reactions recorded as the least satisfied constraints over time. Once the loop has been found ((v_1, v_2, v_3) in this

case), its fluxes will be corrected according to mass balance by just adding a common term in such a way that the reaction carrying the smallest flux (in absolute value) is inverted. In this case, the update inverts flux v_1. After this, the algorithm will re-start looking for a feasible chemical potential vector. The output for this part would look as follows:

```
Exceedingly large times: searching for loops
searching among 3 reactions
checking for loops large 2
checking for loops large 3
loop of size 3 among
reaction 0 v_0=-1
reaction 1 v_1=2
reaction 2 v_2=2
correct them by 1.1
v_0=0.1
v_1=0.9
v_2=0.9
reaction 3 has flux v_3=0.9 but the free energy is G_3=2
from g_2=3 to g_2=3.1
from g_3=5 to g_3=4.9
reaction 3 has flux v_3=0.9 but the free energy is G_3=1.8
from g_2=3.1 to g_2=3.2
from g_3=4.9 to g_3=4.8
reaction 3 has flux v_3=0.9 but the free energy is G_3=1.6
from g_2=3.2 to g_2=3.3
from g_3=4.8 to g_3=4.7
reaction 3 has flux v_3=0.9 but the free energy is G_3=1.4
from g_2=3.3 to g_2=3.4
from g_3=4.7 to g_3=4.6
reaction 3 has flux v_3=0.9 but the free energy is G_3=1.2
from g_2=3.4 to g_2=3.5
from g_3=4.6 to g_3=4.5
reaction 3 has flux v_3=0.9 but the free energy is G_3=1
from g_2=3.5 to g_2=3.6
from g_3=4.5 to g_3=4.4
reaction 3 has flux v_3=0.9 but the free energy is G_3=0.8
from g_2=3.6 to g_2=3.7
from g_3=4.4 to g_3=4.3
reaction 3 has flux v_3=0.9 but the free energy is G_3=0.6
from g_2=3.7 to g_2=3.8
from g_3=4.3 to g_3=4.2
reaction 3 has flux v_3=0.9 but the free energy is G_3=0.4
from g_2=3.8 to g_2=3.9
from g_3=4.2 to g_3=4.1
reaction 3 has flux v_3=0.9 but the free energy is G_3=0.2
from g_2=3.9 to g_2=4
from g_3=4.1 to g_3=4
reaction 3 has flux v_3=0.9 but the free energy is G_3=-0
from g_2=4 to g_2=4.1
```

```
from g_3=4 to g_3=3.9
 chemical potentials:
10   4.1   3.9
 fluxes:
1   0.1   0.9   0.9   1
```

We have finally obtained the mass-balanced flux configuration defined by $(u, w) = (1, 1)$ and $\mathbf{v} = (0.1, 0.9, 0.9)$ with chemical potentials $\boldsymbol{\mu} = (10, 4.1, 3.9)$, corresponding to thermodynamic feasibility.

We remind that in presence of a cycle in the N-dimensional flux configuration $\mathbf{v}$, by definition there exist N numbers $k_i \geq 0$ (not all zero) such that

$$\sum_{\alpha} \mathrm{sign}(v_i) k_i S_{\alpha,i} = 0 \quad \forall \alpha \ . \tag{1}$$

Now, if we consider the configuration $\mathbf{v}'$ defined as

$$v'_i = v_i + \lambda k_i \mathrm{sign}(v_i) \ , \tag{2}$$

it is easy to see that $\mathbf{v}$ and $\mathbf{v}'$ satisfy the same mass balance equations for constant λ. This means that starting from a mass-balanced configuration the above procedure yields another mass-balanced configuration.
